# Whole-Genome Sequencing Analysis of Sapovirus Detected in South Korea

**DOI:** 10.1371/journal.pone.0132328

**Published:** 2015-07-10

**Authors:** Hye Lim Choi, Chang-Il Suh, Seung-Won Park, Ji-Young Jin, Han-Gil Cho, Soon-Young Paik

**Affiliations:** 1 Department of Microbiology, College of Medicine, The Catholic University of Korea, 222 Banpo-daero, Seocho-gu, Seoul, 137–701, Republic of Korea; 2 Department of Medical Consilience, 152, Dankook University, Jukjeon-ro, Suji-gu, Yongin-si, Gyeonggi-do, 448–701, Republic of Korea; 3 Division of Biotechnology, Catholic University of Daegu, Daegu, 712–702, Republic of Korea; 4 Division of Public Health Research, Gyeonggi Province Institute of Health and Environment, Suwon, Republic of Korea; Centers for Disease Control and Prevention, UNITED STATES

## Abstract

Sapovirus (SaV), a virus residing in the intestines, is one of the important causes of gastroenteritis in human beings. Human SaV genomes are classified into various genogroups and genotypes. Whole-genome analysis and phylogenetic analysis of ROK62, the SaV isolated in South Korea, were carried out. The ROK62 genome of 7429 nucleotides contains 3 open-reading frames (ORF). The genotype of ROK62 is SaV GI-1, and 94% of its nucleotide sequence is identical with other SaVs, namely Manchester and Mc114. Recently, SaV infection has been on the rise throughout the world, particularly in countries neighboring South Korea; however, very few academic studies have been done nationally. As the first whole-genome sequence analysis of SaV in South Korea, this research will help provide reference for the detection of recombination, tracking of epidemic spread, and development of diagnosis methods for SaV.

## Introduction

Sapovirus (SaV) is the one of the etiological agents of human gastroenteritis and is named after the Japanese city Sapporo, where it was first discovered [1]. It is an important cause of gastroenteritis in young children and adults, and can induce symptoms such as diarrhea, vomiting, and fever [2, 3]. Its transmission routes are person-to-person (fecal–oral), through aerosols, or through contaminated water or foods [4].

SaV is an RNA virus with a non-segmented, positive-sense, single-stranded RNA molecule of approximately 7.3–7.5 kb. It belongs to the family Caliciviridae, which also includes norovirus [5, 6]. Phylogenetic analysis based on capsid protein (VP1) nucleotide sequences can divide this genus into 5 genogroups (GI–GV). Further analysis of 4 human SaV genogroups has led to their subdivision into 16 genotypes (GI.1–GI.7, GII.1–GII.7, GIV, and GV) [5–7]. Genogroups GI, GII, GIV, and GV can cause severe infection in humans, while GIII infects pigs [8]. GII and GIII genogroups have 2 ORFs, and the others have 3 each [9–11]. ORF1 encodes nonstructural proteins and the capsid protein VP1, but the roles of ORF2- and ORF3-encoded proteins have not been clearly defined [6, 12]. For human SaV strains which were not cultivable through cell culture, molecular studies including characterization of the infectious cycle of the virus were limited. The detection system for SaV with reverse transcription-polymerase chain reaction (RT-PCR) analysis needs to be highly sensitive and accurate [13–15]. The purpose of this study was to analyze and present, for the first time, the full-length genome sequence of a SaV in South Korea. Phylogenetic analysis was performed for comparison with genotypes which have already been reported. We expect the data acquired from whole-genome sequencing to be useful not only for research in molecular biology, but also for basic epidemiologic analyses such as tracking of international spread.

## Materials and Methods

### Ethics statement

The stool sample was provided by Waterborne Virus Bank (WAVA). Due to issues concerning difficulties in tracking the exact records of the patient from the donor hospital, informed consent from the parent of the child participant could not be acquired. The Institutional Review Board reviewed and approved the use of this sample for the purpose of research as this study does not affect the patient. All of the experimental work and sample collections were supervised by the Catholic Medical Center Office of Human Research Protection Program (CMC OHRP) of South Korea (approval no. MC14SISI0096).

### Sample preparation and viral RNA extraction

A SaV-positive stool sample, obtained from a female infant who presented with fever and diarrhea, was obtained from the Waterborne Virus Bank (WAVA, Seoul, South Korea). The stool sample was stored at −70°C until RNA extraction. The frozen stool sample was thawed and diluted with 10% with phosphate-buffered saline (PBS), after which it was centrifuged. Viral RNA of SaV was extracted from 140 μL of supernatant using a QIAamp Viral RNA mini kit (Qiagen, Hilden, Germany) according to the manufacturer’s instructions. Isolated RNA was stored at −70°C until further use.

### Reverse transcription (RT) polymerase chain reaction

For the detection of SaV, RT-PCR was performed with the OneStep RT-PCR Kit (Qiagen) using SV-F11 and SV-R1 primers (Table 1). To analyze the whole-genome sequence of SaV, 10 more primer pairs were newly designed based on the Manchester strain (GenBank accession no. X86560). RT-PCR was performed with a S1000 thermal cycler (Bio-Rad, Hercules, CA, USA), and the steps comprised RT (50°C for 30 min), initial PCR activation (95°C for 15 min), 39 cycles of 3-step cycling (94°C for 30 s, 52°C–55°C for 30 s, and 72°C for 1 min), and final extension (72°C for 10 min). All RT-PCR products were examined by electrophoresis in ethidium bromide-stained 2% agarose gels.

**Table 1:** Primers used in this study.

Primer	Sequence (5’→3’)	Location	Size (bp)	Polarity
KSV-F1	GTG ATT GGT TRG ATG GYT TCC	1–21	847	+
KSV-R1	CYA CDA GTG CTG TCA TGG TG	828–847		-
KSV-F2	ACC ATC ATC CTY CAA CAA CAC AA	784–811	878	+
KSV-R2	TCA CAA TTD ART GGG AAS GGT G	1640–1661		-
KSV-F3	TGC ATA TGG GAT GAR TTT GAT GTS	1567–1590	797	+
KSV-R3	GTG TGS TKG TGG WAA ACA TGG A	2342–2363		-
KSV-F4	CMA TGT TYA CCA CCA SYA CAC	2267–2287	842	+
KSV-R4	ATC YCG CAC RCC ACC ACG A	3090–3108		-
KSV-F5	ACA TGA SWG TCA ATG ACT TCC TMA	2945–2968	706	+
KSV-R5	GSA RRC CCT TCC AKT GAA ATT CA	3628–3650		-
KSV-F6	TGA ATT TCA MTG GAA GGG YYT SC	3633–3655	748	+
KSV-R6	TTG TGT GGA RTC CCY TTT DGA R	4359–4380		-
KSV-F7	YTC HAA ARG GGA YTC CAC ACA A	4359–4380	821	+
KSV-R7	TGC CCT CCA TCT CAA ACA CTA	5159–5179		-
KSV-F8	ATG GAM AAT GGK GTK TCA CCW G	5857–5878	632	+
KSV-R8	AGC CAG TGT GGC TGT GA	6473–6488		-
KSV-F9	GAC TTT GAC ACY AGT GGY TTT GC	6379–6401	670	+
KSV-R9	CCA TTR ATG GAG AGG TCY CG	7029–7048		-
KSV-F10	ATA GTR TAC CAG CAG CGY CAG	6921–6941	511	+
KSV-R10	GSR GGR ACG GYG ACA ATC	7414–7431		-
*SV-F11	GCY TGG TTY ATA GGT GGT AC	5098–5117	781	+
*SV-R1	CWG GTG AMA CMC CAT TKT CCA T	5857–5878		-
5’-SV-PR	CAG ATT TGA ATA CAC CTC CAC TA	842–864		-
5’-SV-F1	CCA TTA CAG AAC CCA TTA	659–676	483	+
5’-SV-R1	GAA ACA ATG CAC CTT CTT	260–277		-
5’-SV-F2	CAG TAC AAC AAG AAA TGG	704–721	385	+
5’-SV-R2	AGG AGA CCC TCC TCG ACG AA	205–224		-
3’-Oligo (dT)-anchor	CAA TGA GGT TAT GGC TTT GGA ACT TTTTTT TTT TTT TT			-
3’-Anchor-R	CAA TGA GGT TAT GGC TTT GGA AC			-

*Molecular epidemiology and phylogenetic analysis of Sapporo-like viruses.

The primers were based on the Manchester strain (GenBank accession no. X86560)[13].

### Determination of the 5’ and 3’–ends of the SaV genomic RNA

To determine the 5'-end of the SaV genomic RNA, RACE (Rapid-Amplification of cDNA Ends) was performed with the 5'-Full RACE Core Set Kit (Takara Bio Inc., Ohtsu, Japan). The first cDNA strand was synthesized through reverse transcription from target mRNA using 5' end-phosphorylated RT Primer (5’-SV-PR, Table 1), after which it was treated with RNAse H to remove hybrid RNA and with RNA Ligase to form circularized single-strand cDNA or concatemers. To amplify the product, the first PCR reaction was performed using 5’-SV-F1 and 5’-SV-R1 primers under the following conditions: 94°C for 3 min, followed by 25 cycles each of 94°C for 30 sec, 56°C for 30 sec and 72°C for 5 min. Then, the second PCR reaction was conducted with 5’-SV-F2 and 5’-SV-R2 primers through 30 cycles of 3-step cycling (94°C for 30 sec, 56°C for 30 sec and 72°C for 5min).

To attain the exact sequence for the 3'-end of the SaV genomic RNA, cDNA was synthesized using RT reaction performed with 3'-end poly A tail-based 3’-Oligo (dT)-anchor primer (Table 1). The second PCR reaction was conducted using the SV-10F and 3’-anchor-R primers (Table 1) under the following conditions: 30 cycles of 3-step cycling (98°C for 10 sec, 56°C for 30 sec and 72°C for 1min) and 72°C for 7min.

### Cloning and sequencing of the complete genome

All PCR products obtained using 13 primer pairs were extracted from 2% agarose gels using HiYield Gel/PCR DNA Fragments Extraction Kit (RBC, Taipei, Taiwan) and were cloned into pGEM-T easy vectors (Promega, Madison, WI, USA). Transformed *Escherichia coli* DH5α-competent cells (RBC) were selected from Luria-Bertani (LB) agar plate (Duchefa, Haarlem, Netherlands) containing 40 mg/mL X-gal, 0.1 mM isopropyl-β-d-thio-galactoside, and 50 mg/mL ampicillin at 37°C for 16–18 h. Selected clones were inoculated in LB broth and incubated overnight in a shaking incubator (37°C, 200 rpm, IS-971R, Jeiotech, Daejeon, South Korea). Plasmid DNA was purified using the HiYield Plasmid Mini Kit (RBC) and sequenced (Cosmo Genetech, Seoul, South Korea). The sequencing results were analyzed using NCBI’s BLAST.

### Phylogenetic analysis

Comparative sequence analysis, including sequence alignments and estimation of genetic distances, was performed with Clustal W using the Molecular Evolutionary Genetic Analysis software (MEGA soft version 6.0) [16]. Phylogenetic trees were constructed using the neighbor-joining method in MEGA 6 [17].

## Results

The SaV RNA was extracted from a stool sample collected and provided by the Waterborne Virus Bank (WAVA, Seoul, South Korea). The isolated SaV strain, designated as ROK62, had a total length of 7429 nucleotides (nt). The complete genome sequence of ROK62 was deposited in GenBank under accession no. KP298674. Its 5ʹ-UTR was12 nt long, and 3ʹ-UTR was 81 nt long. Its total length was found to be the same as that of the Mc114 virus and was 2 nt shorter than that of the Manchester virus. The location and length of ORFs and VP regions were found to be as follows: ORF1, 13–6855 (6843 nt); ORF2, 6852–7349 (498 nt); ORF3, 5180–5665 (486 nt); and VP1, 5170–6852 (1683 nt).

**Fig 1:**
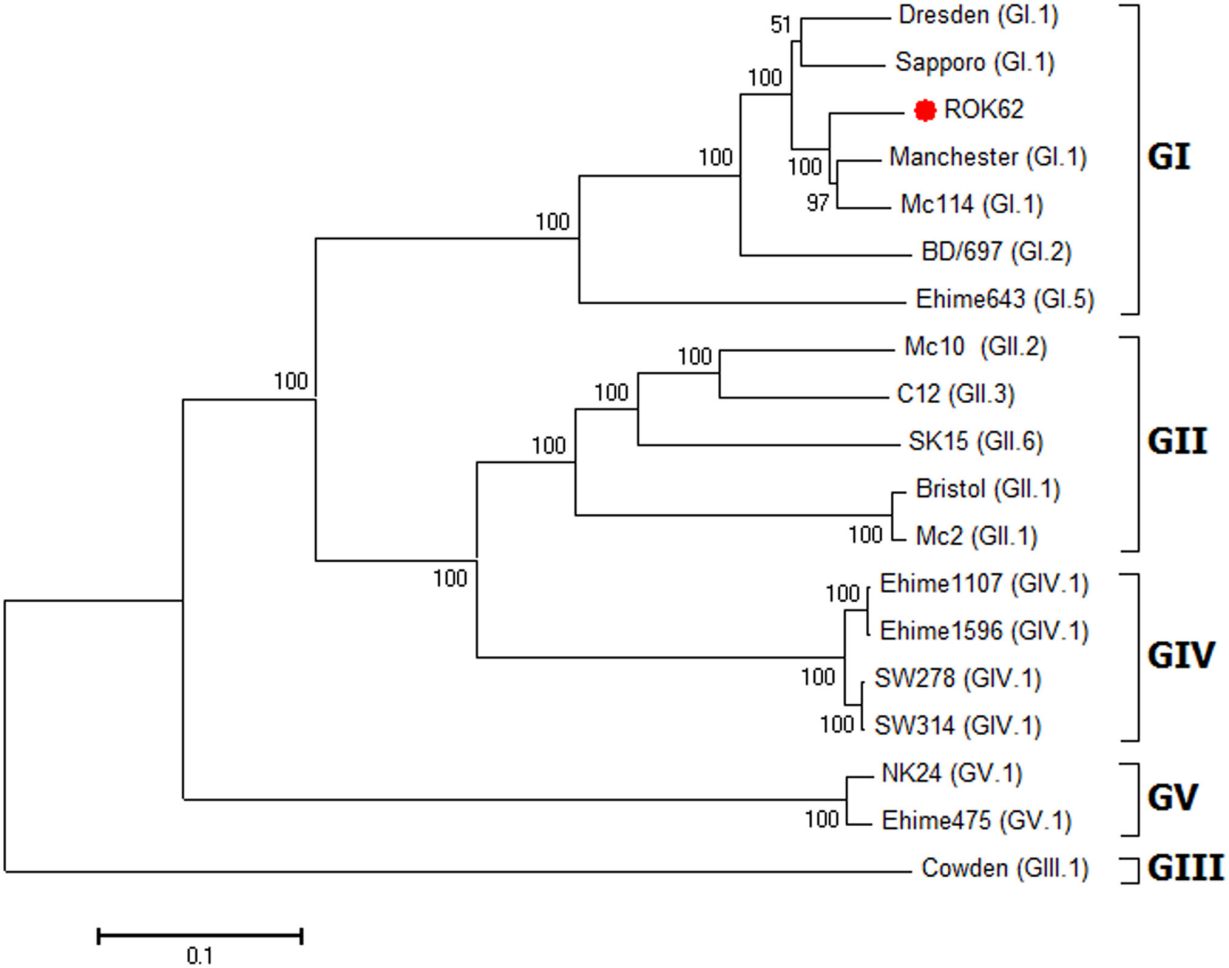
Phylogenetic tree of sapoviruses based on whole-genome sequences. The numbers associated with each branch indicate the bootstrap values for the genotype. The neighbor-joining method in MEGA was used to construct the trees. Statistical significance for the grouping was obtained when bootstrap values were greater than 95. The scale shows nucleotide substitution per site. The genogroup and genotype of each strain was indicated with strain name, inside ( ). The GenBank accession numbers of the reference strains are as follows: Bristol, AJ249939; BD/697, GQ261222; C12, AY603425; Cowden, AF182760; Dresden, AY694184; Ehime475, DQ366344; Ehime643, DQ366345; Ehime1107, DQ058829; Ehime1596, DQ366346; Manchester, X86560; Mc114, AY237422; Mc2, AY237419; Mc10, NC010624; NK24, AY646856; SK15, AY646855; SW278, DQ125333; SW314, DQ125334; Sapporo, HM002617.

In the phylogenetic analysis, ROK62 sequences were aligned and compared with other reported SaV sequences. In the phylogenetic tree, ROK62 was classified under the GI genogroup, closely resembling the Manchester virus and the Mc114 virus, which are SaV GI-1 members (Fig 1). Similarities with the Manchester strain (GenBank accession number X868560) was confirmed using Basic Local Alignment Search Tool (BLAST) analysis, which revealed an identity of 94% (highest similarity), Max scores, total scores, and query coverage values were also determined, and their values were 11271, 11271, and 100%, respectively, for ROK62 and the Manchester virus. ROK62 showed 94% identity with the Mc114. The identity was determined using whole-genome sequence BLAST (Table 2). All identity results were obtained at a query coverage rate greater than 99%.

**Table 2:** SaV, ROK62, BLAST results.

**Description**	**Max score**	**Total score**	**Query cover**	**E value**	**Identity**	**Accession number**
**Sapporo virus-Manchester polyprotein and hypothetical proteins, genomic RNA**	11271	112713	100%	0.0	94%	X86560
**Sapovirus Mc114, complete genome**	11099	11099	99%	0.0	94%	AY237422
**Sapovirus N21, complete genome**	11021	11021	100%	0.0	93%	AY237423
**Sapovirus Hu/GI/Sapporo/MT-2010/1982, complete genome**	9806	9806	100%	0.0	90%	HM002617
**Sapovirus Hu/Dresden/pJG-Sap01/DE, complete genome**	9607	9607	99%	0.0	90%	AY694184
**Sapovirus NongKhai-50/Thailand, complete genome**	9581	9581	100%	0.0	90%	AY646853
**Sapovirus Chanthaburi-74/Thailand, complete genome**	9437	9437	100%	0.0	90%	AY646854

**Fig 2:**
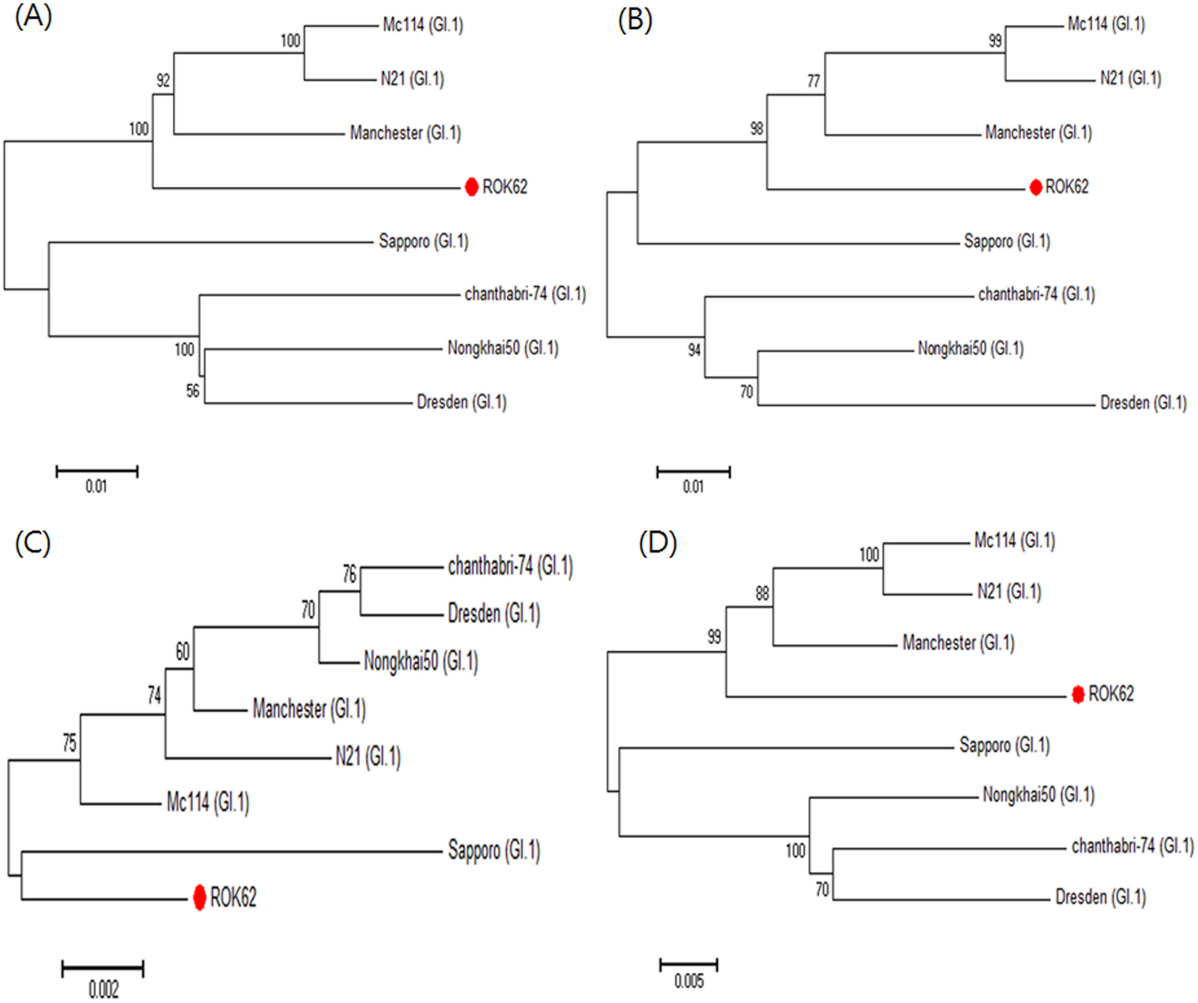
Phylogenetic trees of sapoviruses based on the sequences of (A) ORF1, (B) ORF2, (C) ORF3, and (D) VP1. The trees were constructed with the neighbor-joining method as in Fig 1. The analysis was carried out for 7 isolates of GI genogroup. Red circles in the trees indicate ROK62. The genogroup and genotype of each strain was indicated with strain name, inside (). The GenBank accession numbers for the reference strains are as follows: Mc114, AY237422; N21, AY237423; Manchester, X86560; Sapporo, HM002617; Chanthabri-74, AY646854; Nongkhai50, AY646853; Dresden, AY694184.

Even though phylogenetic analysis of ORF3 showed that ROK62 is close to the Sapporo strain, sequence identities with ORF1 (99.93%), ORF2 (99.93%), ORF3 (99.99%) and VP1 (99.95%) showed the closest relationships with the Manchester strain (Fig 2).

## Discussion

SaV is one of the important causal agents of acute gastroenteritis worldwide. It mostly infects children but can also infect adults [18], and can occur in during any season [19, 20].

Although the occurrence rate of SaV in South Korea reported in 2012 was not high (0.1%) [21], it has been increasing globally. For example, the rate of SaV-positive gastroenteritis outbreaks was reported to be as high as 8% according to studies in 2000–2012 in Japan [22]. Moreover, there have been steady occurrences of SaV infections in other Asian countries, including China, Thailand, Taiwan, and Hong Kong. SaV infections have also been reported in European countries such as Germany, Sweden, and the Netherlands, where the rates of SaV-positive gastroenteritis outbreaks were in the range of 1.3–4% [23].

This is the first study to determine the whole genome sequence of SaV from a patient with acute gastroenteritis in South Korea. The SaV strain, ROK62, which was detected in South Korea, belongs to GI-1 and showed no intra- or inter-genogroup recombination of the nonstructural protein-encoding region and the VP1-encoding region. ROK62 is very similar to the Sapporo (Hu/GI/Sapporo/MT-2010/1982, HM002617) strain, the first prototype of which was reported from an outbreak in Sapporo, Japan, in 1982 [24–27). Phylogenetic analysis showed that the strain which shows the most resemblance is the Manchester strain (Sapporo virus-Manchester/UK, X86560), which was detected in the United Kingdom in 1993 and was the first SaV to have its complete genome sequenced [28, 29]. The genomic organization of ROK62, including the location and length of ORFs, VP1, and VP2, was the same as that of the Manchester strain. The Mc114 (Sapovirus Mc114/JPN, AY237422) and N21 (Sapovirus N21/THA, AY237423) strains were also very similar. Periodic monitoring of SaV is needed to keep track of the dynamic changes of genogroups and genotypes, as predominant genogroups and genotypes of vast diversity have been reported in the same geographical area [30–32).

Phylogenetic analysis of the currently circulating SaVs is necessary in order to remain updated regarding the rapid evolution of SaV strains. Around 2007, GIV.1 was the predominant SaV strain detected in Japan, Canada, the United States, and Europe, and therefore surveillance was considered important not only at the national level but also at the international level [32–36]. This underscores the importance of international cooperation in the form of information exchange among nations, in addition to national surveillance, for the prevention of epidemics. Through comparative using more data concerning whole-genome sequencing from South Korea and neighboring countries, the development of detection kits for discovering the current predominant strains and for the prediction of future predominant strains can be developed. Therefore, we surmise that this study will not only prove valuable for basic epidemiological research but also for the promotion of public health.
